# Novel Regulatory Factors and Small-Molecule Inhibitors of FGFR4 in Cancer

**DOI:** 10.3389/fphar.2021.633453

**Published:** 2021-04-26

**Authors:** Yanan Liu, Canwei Wang, Jifa Li, Jiandong Zhu, Chengguang Zhao, Huanhai Xu

**Affiliations:** ^1^ Affiliated Yueqing Hospital, Wenzhou Medical University, Wenzhou, China; ^2^ School of Pharmaceutical Sciences, Wenzhou Medical University, Wenzhou, China

**Keywords:** FGFR4, FGF19, cancer, activator, inhibitor

## Abstract

Fibroblast growth factor receptor 4 (FGFR4) is a tyrosine kinase receptor that is a member of the fibroblast growth factor receptor family and is stimulated by highly regulated ligand binding. Excessive expression of the receptor and its ligand, especially FGF19, occurs in many types of cancer. Abnormal FGFR4 production explains these cancer formations, and therefore, this receptor has emerged as a potential target for inhibiting cancer development. This review discusses the diverse mechanisms of oncogenic activation of FGFR4 and highlights some currently available inhibitors targeting FGFR4.

## Introduction

FGFR1–4 and FGFR5 comprise the fibroblast growth factor receptor (FGFR) family (Wang and Ding, 2017). Among these members, FGFR1–4 are typical tyrosine kinase receptors, including a cell surface segment, a one-way cross-membrane section, and a protein–tyrosine kinase domain inside the membrane. FGFR5 is also called FGFRL1, and it differs from the others in that it is missing the intracellular kinase domain (Regeenes et al., 2018). In the process of FGF–FGFR binding, the receptor and the ligand combine to form a dimer stimulated and autophosphorylation complex leads to downstream pathways, including protein serine–threonine kinase (AKT), mitogen-activated protein kinase (MAPK), and signal transducer and activator of transcription 3 (STAT3)-activated pathways (Helsten et al., 2016). Current researchers have determined that the FGFR protein family participates in the generation of tumor cells, angiogenesis, immigration, differentiation, aggression, and drug resistance (Haugsten et al., 2010).

Among the FGFR family members, the role of FGFR4 in cancer has been expounded on by only a few studies. Herein, this review discusses the characteristics of FGFR4 signaling in tumor progression and features some small molecular inhibitors that target FGFR4, intending to increase our understanding of this pathway.

## FGFR4 in Cancer

**FIGURE 1:**
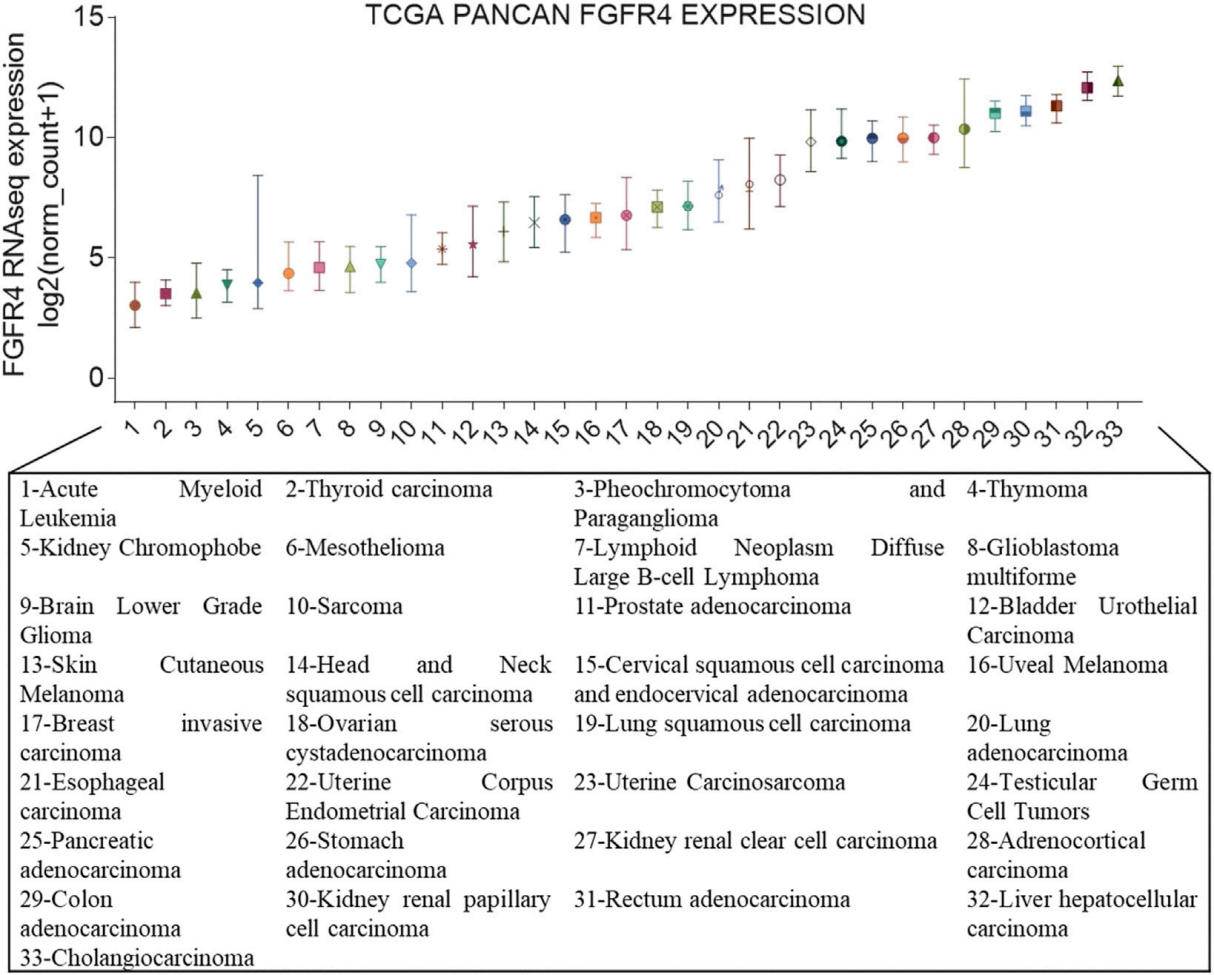
FGFR4 gene expression in cancer. Expression of FGFR4 in different cancer types from The Cancer Genome Atlas (TCGA). On the *x*-axis are the different cancer types in TCGA, and the *y*-axis depicts gene expression RNAseq of FGFR4 (IlluminaHiSeq), unit: log2 (norm_count+1).

Genetic aberrations in FGFR4 are prevalent among various types of cancer like breast cancer, pancreatic cancer, and especially hepatocellular carcinoma (HCC) (Figure 1), and these aberrations are associated with poor prognoses (Shah et al., 2002; Sawey et al., 2011; Jain and Turner, 2012). The irregular expression of the FGFR4 pathway may be induced by gene amplification, posttranscriptional errors (Helsten et al., 2016), FGFR4 mutations (Futami et al., 2019), translocations, isoform switching, alternative splicing of FGFR4 (Kwiatkowski et al., 2008), and overexpression of specific ligands in cancer or stromal cells (Miura et al., 2012).

Regarding the aberrations and abnormalities in the FGFR4 gene, except for point mutations, gene fusions, and splice variations, one crucial genetic mutation is the single nucleotide polymorphism (SNP). SNPs can exist in various regions of DNA and can affect the production of transcription factors, translation, and gene expression. Recently, the popularity and widespread use of SNP analysis platforms have made the identification of individual SNPs more convenient (Tsay et al., 2019). The SNPs from FGFR4 have been recognized as an essential participant in cancer occurrence and were associated with prognosis in patients. Examples are FGFR4 rs351855 in HCC (Sheu et al., 2015), FGFR4 SNP rs2011077 with rs1966265 in urothelial cell carcinoma (Tsay et al., 2019), and FGFR4 rs2011077 and rs1966265 in oral squamous cell carcinoma. FGFR4 can alter the production of relative transcript factors, which influences subsequent translation and gene expression (Su et al., 2018) and is directly related to patient survival (Chou et al., 2017).

## FGFR4-Specific Ligand: FGF19

**FIGURE 2:**
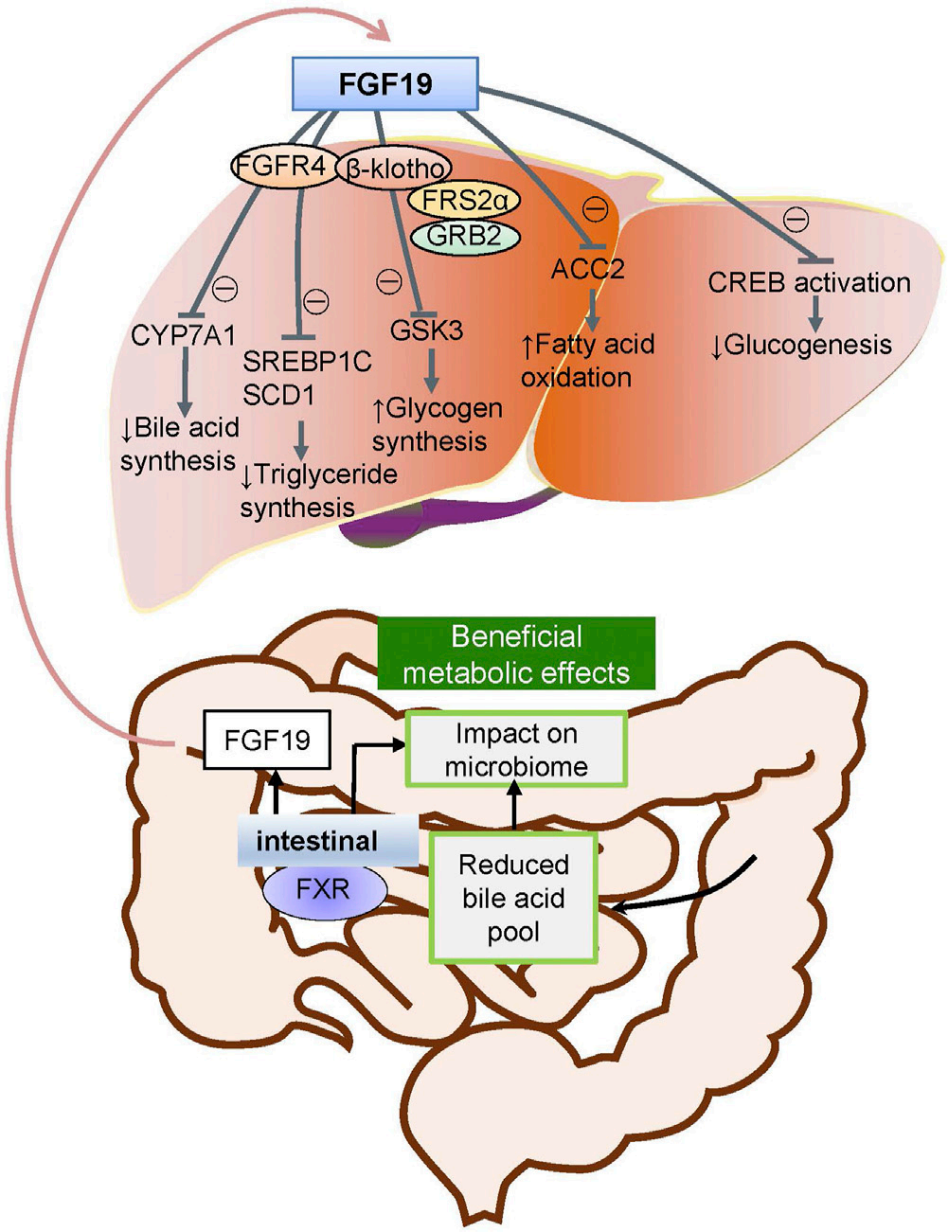
Metabolic pathways of FGF19 during normal conditions. Under normal conditions, FGF19 is secreted from the intestinal canal and enters into the enterohepatic circulation into the liver and subsequently binds to the specific receptor activating the FGF19/klotho/FGFR4 pathways. This results in the metabolic regulation of glucogenesis, fatty acid oxidation, glycogen synthesis, triglyceride synthesis, and bile acid synthesis.

**FIGURE 3:**
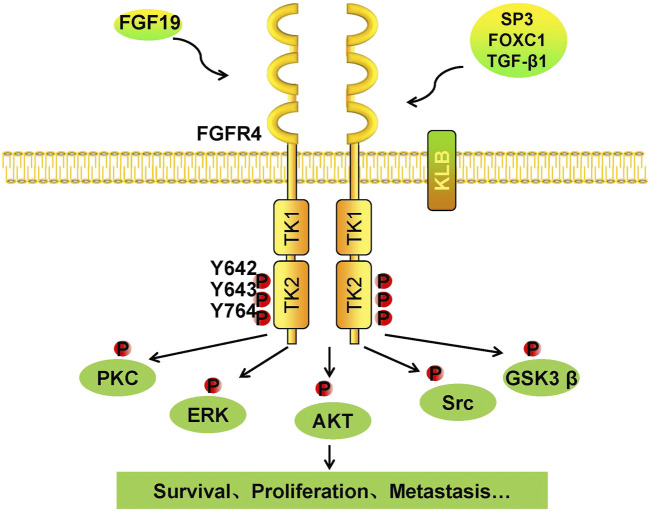
Tumorigenic mechanism of the FGF19/klotho/FGFR4 signaling pathways. Once FGF19 binds to FGFR4 with klotho, tyrosine residues in the intramembrane TK2 domain are phosphorylated, and FGFR4 is activated. Phosphorylated FGFR4 activates downstream kinases, including PKC, ERK, AKT, Src, and GSK3β. Cells respond to these activated kinases, and survival, proliferation, and metastasis result.

FGFs function mainly in paracrine and autocrine metabolism (Li, 2019). However, FGF19 subfamilies, including FGF19, FGF21, and FGF23, act as endocrine factors or hormones that bind to specific receptors. FGF19 plays an essential role in metabolism under ordinary physiological conditions (Lin and Desnoyers, 2012). FGF19 subfamily proteins affect the enterohepatic circulation of bile involved in glucose and lipid metabolism and maintain homeostasis phosphorus and vitamin D3 (Dolegowska et al., 2019). Under normal circumstances, the intestinal tract secretes FGF19, and it binds to FGFR4 on liver cells through the hepatoenteral circulation to regulate metabolism (Liu et al., 2020) (Figure 2). In disease states, FGF19 might be crucial for the development and progression of multiple cancers. The specific binding of ligand FGF19 combined with co-receptor β-klotho activated FGFR4. FGF19-mediated activation of the phosphatidylinositol-3-kinase (PI3K)/AKT, MAPK, STAT3, and epithelial–mesenchymal transition (EMT) pathways might take part in the malignancy (Figure 3) (McKinnon et al., 2018; Xin et al., 2018).

**FIGURE 4:**
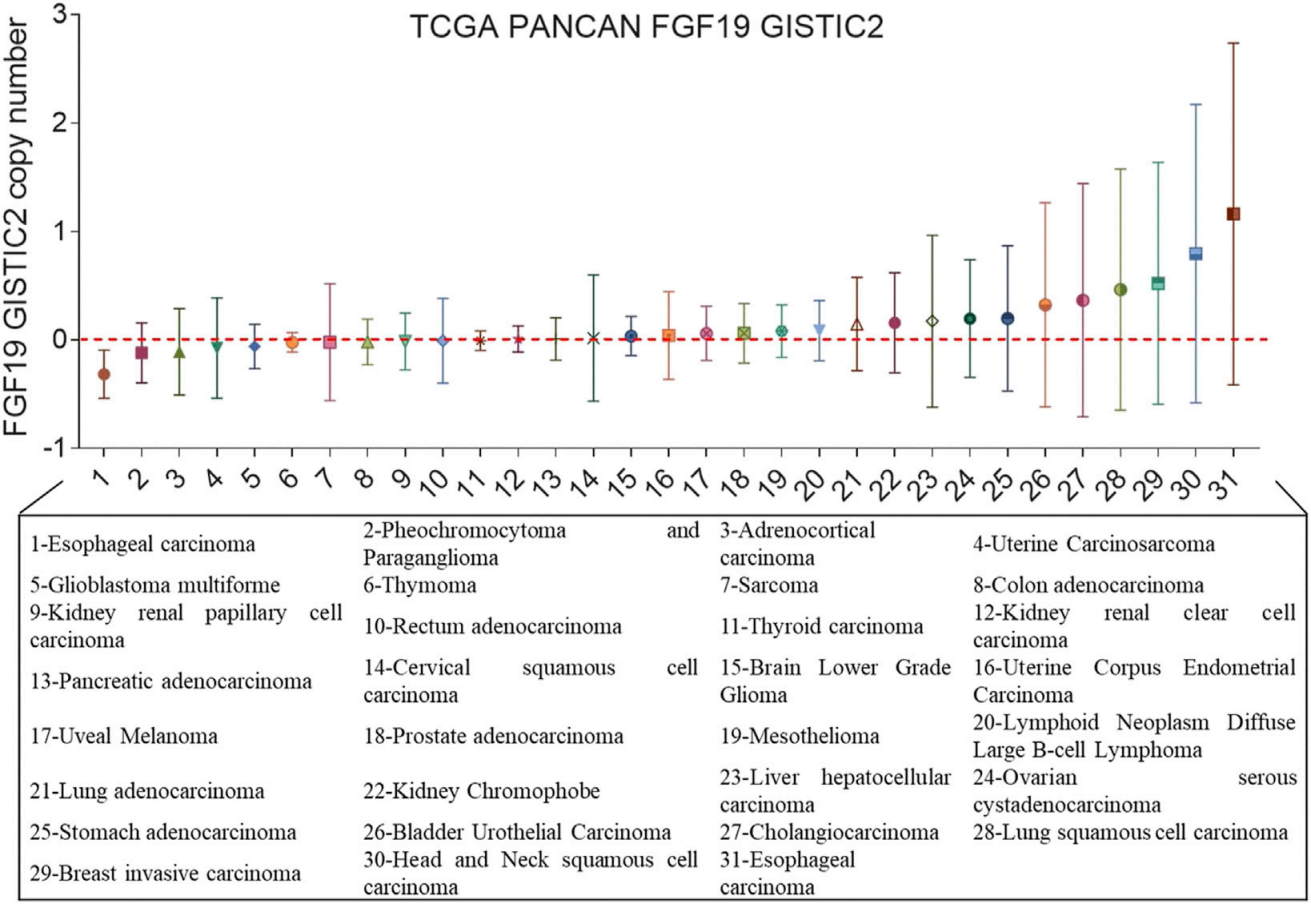
FGF19 gene copy number in cancer. The gene copy number of FGF19 in different cancer types from The Cancer Genome Atlas (TCGA). On the *x*-axis, there are 31different TCGA tumor types, and on the *y*-axis, there are the gene copy numbers of FGF19; unit: Gistic2 copy number.

The FGF19 gene is located in 11q13.3, an amplified section of which is usually found in human HCC (Wu and Li, 2011). An orthotropic transplantation study confirmed that the transplanted hepatocytes overexpressing FGF19 developed tumors (Schwartz, 2011). A monoclonal FGF19-blocking antibody was created to prove the function of FGF19 in cancer development. When examining *in vivo* and chemically induced liver tumor models, it was observed that the FGF19 antibody suppressed tumor growth (Desnoyers et al., 2008). FGF19 gene amplification is common in several types of cancers, such as lung squamous cell carcinoma (Lang and Teng, 2019), breast cancer (Zhao et al., 2018), and esophageal cancer (Liu et al., 2020) (Figure 4). It is suggested that a potent approach to treating different types of cancer would involve targeting the FGF19 gene to silence it.

## Other Regulatory Factors of FGFR4

Identifying the active mechanisms of FGFR4 can be an optimal strategy to develop new therapeutic inhibitors (Table 1). MicroRNAs (miRNAs) play an essential role in developing tumors because they can inhibit the transcription of corresponding target genes (Ueda et al., 2010). Recent research has claimed that miR-7-5p could bind to FGFR4 3’-UTR directly (Tian et al., 2020). It has been demonstrated that miR-491-5p suppresses tumor growth in certain cancers and can indirectly inhibit FGFR4, thus reducing the SNAIL level and weakening EMT-induced tumor migration (Yu et al., 2018). Another study demonstrated that overexpressed miR-29c-3p reduced the secretion of KIAA1199, a cell migration–inducing protein. Subsequently, suppressing the EGFR and FGFR4/AKT pathways’ excitation was ultimately harmful to EMT (Wang et al., 2019).

**TABLE 1:** Novel activators of FGFR4 in cancer cells.

Activators	Category	Mechanism	Functions
MiR-491-5p	microRNA	Binding to the FGFR4 RNA	Suppressed EMT and tumor metastasis
MiR-29c-3p	microRNA	Decreased expression of KIAA1199, subsequently suppressed the activation of the FGFR4/Wnt/β-catenin	Harmful to the EMT
IL-1β	Pro-inflammatory cytokine	Inhibited β-klotho expression, thus inhibiting FGF19/FGFR4-induced Erk1/2 activation	Promote cell growth, migration, and invasion capacity
Sp1	Transcription factors	Binding to promoter location, repress FGFR4 gene activity	Repress myogenic differentiation
Sp3	Transcription factors	Binding to promoter location active FGFR4 gene activity	Promote myogenic differentiation
FOXC1	Transcription factors	FOXC1 directly binds its target genes ITGA7 and FGFR4 and activates their expression	Promote lymphatic vessel formation, angiogenesis, and metastasis
TGF-β1	Cytokines	Induce FGFR4 expression through the ERK pathway	Promote EMT and cancer dissemination

Interleukin-1β (IL-1β) acts as an essential pro-inflammatory cytokine mediating the innate immune response. It helps the host resist the invasion of microorganisms and is beneficial for body repair (Dinarello, 2011). Zhao et al. found that among the inflammatory cytokines released in response to lipopolysaccharide (LPS), an immune response resulted only when IL-1β specificity restricted the expression of β-klotho. After losing its co-receptor, FGF19 cannot successfully combine with FGFR4. Thus, IL-1β restrained FGF19/FGFR4-induced MAPK phosphorylation and tumor generation (Zhao et al., 2016b). The result suggests that inflammatory cytokines, especially IL-1β, can influence the activation of the FGF19/FGFR4 signaling pathway in the tumor microenvironment.

Specificity protein (Sp) transcription factors (TFs) play an essential role in promoting cancer. Accumulation of Sp1, Sp3, and Sp4 in cells leads to tumor development in organisms (Suske et al., 2005). One study showed that Sp TFs participate in the occurrence and development of a tumor, and they also support tumor resistance to drugs. Sp1, Sp3, and Sp4 are known as no oncogene addiction (NOA) genes and have become relevant drug targets (Safe et al., 2018). Another research demonstrated that the Sp TFs control the FGFR family’s activation (Cavanaugh and DiMario, 2017). Mutation analysis investigated the three Sp binding sites on the FGFR4 promoter, and chromatin immunoprecipitation and electromobility shift assays revealed that Sp3 binding occurred at the location of the FGFR4 promoter. After overexpression of Sp1 and Sp3, it was found that Sp1 inhibited FGFR4 expression, but Sp3 promoted FGFR4 expression (Cavanaugh and DiMario, 2017).

Forkhead box C1 (FOXC1), which belongs to the Forkhead box (FOX) transcription factor family, participates in neural crest, ocular, and mesodermal development. It performs a vital role in lymphatic vessel formation, angiogenesis, and metastasis (Elian et al., 2018). The data accumulated over the years demonstrate the unique behavior of FOXC1 in cancer, particularly in basal-like breast cancer (BLBC) (Wang et al., 2018a). Other studies determined that FOXC1 is significantly involved in breast cancer (BRCA) (Sabapathi et al., 2019) and also colon adenocarcinoma (COAD) (Zhang et al., 2020), pancreatic adenocarcinoma (PAAD) (Subramani et al., 2018), and non–small-cell lung cancer (NSCLC) (Chen et al., 2016). Using genetic epistasis analysis, Liu found that FOXC1 attaches to integrin α7 (ITGA7) and FGFR4 and then activates their expression in metastatic colorectal cancer (CRC). FOXC1 overexpression–mediated CRC metastasis can be reverted using an FGFR4 inhibitor (Liu et al., 2018). The research shows that targeting the FGFR4 signaling pathway might be a useful approach that can be used to treat FOXC1-driven CRC metastasis.

Transforming growth factor β1 (TGF-β1) is significantly associated with regulating cell multiplication, differentiation, invasion, and tumor promotion (Troncone et al., 2018). Among animal models, the TGF-β family significantly impacts metabolism and plays a critical role in tumor transformation, proliferation, invasion, extracellular matrix (ECM) production, and immune reaction (Xie et al., 2018). In the tumor microenvironment, TGF-β1 modulates and interferes with EMT progression, is associated with metastasis, and directly binds to membrane receptors TβR-1 and TβR-2 to exert its effect (Fransvea et al., 2009). Few studies have expounded on a correlation between TGF-β and FGFR4. The expression of FGFR4 is correlated with the diagnosis of HCC, which is related to TGF-β expression. The invasive and metastatic effects of TGF-β1 are realized by inducing FGFR4 and its downstream MAPK pathway (Huang et al., 2018).

## FGF19/FGFR4 Activation Results in Resistance to Therapies

The main reason cancer becomes resistant to chemotherapy is that cancer cells have formed antiapoptotic signaling pathways (Zhao et al., 2016a). Substantial studies have shown that the stimulation of the FGFR4 pathway endows cancer with the capacity to resist cancer therapies and chemotherapies (Prieto-Dominguez et al., 2018). A recent study found that breast cancer cell lines can express FGFR4 to gain the ability to resist apoptosis when treated with cyclophosphamide and doxorubicin, while this capacity disappears when the FGFR4 gene is silenced (Andre and Cortes, 2015). FGFR4 overexpression increased Bcl-x expression at the mRNA and protein level through the MAPK cascade, implying that FGFR4 inhibitors (e.g., opposing antibodies) combined with chemotherapeutic drugs should be used for treating FGFR4-overexpressing cancers (Roidl et al., 2009).

Another study showed that drug-resistant cells activate FGFR4 signaling to phosphorylate FGF receptor substrate 2 (FRS2) and then activate downstream MAPK/ERK signaling. Inhibitors that block the FGFR4-FRS2-ERK signaling pathway restrain the glycolytic phenotypes and chemoresistance of resistant cells (Xu et al., 2018). Ahmed et al. (2016) investigated CRC cells that can resist radiotherapy via expression of FGFR4 and discovered that inhibiting FGFR4 can weaken the RAD51-mediated double strain break (DSB) repair, hence attenuating the anti-radiation effect. FGFR4 may be an efficient target for combination therapies to improve radiation response.

**FIGURE 5:**
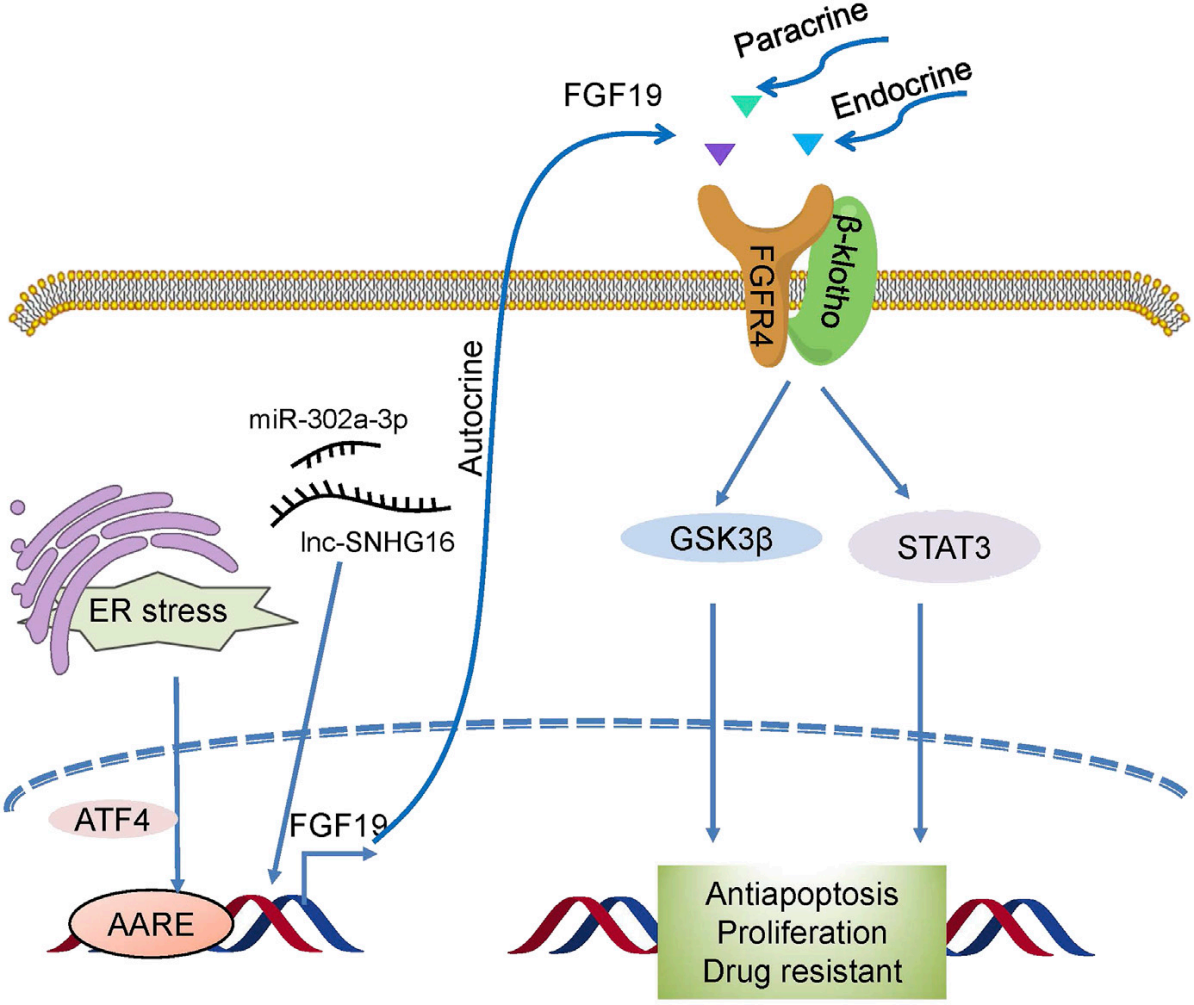
Drug resistance mechanism induced by FGF19/FGFR4. Novel activators act on FGF19/FGFR4, which then activates GSK3β/β-catenin with the STAT3 pathway and confers drug resistance to cells.

FGF19 also plays a crucial role in resistance to therapies (Figure 5). In HCC, overexpression of FGF19 not only promotes EMT by activating the GSK3β/β-catenin and STAT3 pathways (Zhao et al., 2016b) but it also shields HCC cells against endoplasmic reticulum (ER) stress. In liver cancer, ER stress enhanced the transcriptional activation of FGF19 mediated by ATF4, and antiapoptotic ability was observed to increase during ER stress (Teng et al., 2017). Small nucleolar RNA host gene 16 (SNHG16) is a proto-oncogene common to various types of cancer (Gong et al., 2020). One recent study revealed that SNHG16 increased HCC growth and antiapoptosis through the SNHG16/miR-302a-3p/FGF19 pathway (Li et al., 2019). These studies indicate that FGF19 is associated with tumorigenesis, and targeting it may be useful as a form of cancer therapy.

## Novel Small-Molecule Inhibitors of FGFR4 in Cancer

The FGF19/FGFR4 pathway participates in metabolism and maintaining cell processes such as growth and reproduction. Suppression of the expression of FGFR4 and its ligand or the impairment of its downstream activation has been known as the main reason for tumor growth (Liu et al., 2020). It has been reported that FGFR4 possesses three immunoglobulin-like domains (IgI, IgII, and IgIII) outside the membrane structure that is necessary for a particular ligand, which is also the case for the other three FGFRs (Dai et al., 2019). More importantly, unlike FGFR1–3, there are no splice variants of IgIII in FGFR4 (Touat et al., 2015), which may explain why pan-FGFR inhibitors have a low affinity for FGFR4 and suggests that developing selective FGFR4 inhibitors could be an effective therapeutic strategy. Numerous clinical trials have been performed to test several drugs that specifically target FGF19/FGFR4. Thus far, the most investigated approach in anticancer targeting of the FGF19/FGFR4 pathway has been the use of small molecular inhibitors of FGFR4 kinase.

In order to develop effective anticancer inhibitors of FGFR4, great efforts have been made. Several multi-targeting tyrosine kinase inhibitors (TKIs) have been developed for treatment, such as ponatinib (Massaro and Breccia, 2018), dovitinib (Andre et al., 2013), and lucitanib (Hui et al., 2020). Although these inhibitors have good kinase inhibitory activity against FGFR4, their therapeutic effects are limited by their inhibitory effects on other enzymes (Gavine et al., 2012). After analyzing the ATP domain of the FGFR family, a cysteine residue was found. It is possible to design some covalent inhibitors for this residue to inhibit the phosphorylation of FGFR. Pan inhibitors have been successfully reported as covalent inhibitors of FGFR. They all bind the cysteine residue position at position 477, such as TAS-120 (Goyal et al., 2019), FIIN-1 (Zhou et al., 2010), and FIIN-2 (Tan et al., 2014). However, when using these inhibitors, because of targeting both FGFR1 and FGFR3, severely toxic side effects occur in patients with hyperphosphatemia (Wang et al., 2018b). In view of the important fact that the FGFR family shares sequence homology about its kinase domain, developing a selective inhibitor of FGFR4 has been a daunting challenge. In 2015, Hagel et al. reported a selective FGFR4 inhibitor, BLU-9931, by binding the conserved Cys552 in the hinge region of the FGFR4 protein. This may be an effective treatment strategy. Herein, we reviewed the clinical trial results for FGFR4 inhibitors for different cancer types (Table 2).

**TABLE 2:** Overview of novel small-molecule inhibitors of FGFR4 and clinical studies.

Drug	Structural formula	Target(s)	Clinical trial ID	Tumor types	Phase	Status
NVP-BGJ398		Pan-FGFRs inhibitor	NCT01975701	Recurrent resectable unresectable glioblastoma	II	Completed
			NCT03510455	Oncogenic osteomalacia	II	Recruiting
AZD4547		Pan-FGFRs inhibitor	NCT01824901	Non–small-cell lung cancer	I/II	Completed
			NCT01791985	Breast cancer	I/II	Completed
JNJ-42756493 (Erdafitinib)		Pan-FGFRs inhibitor	NCT02421185	Carcinoma, hepatocellular	I/II	Completed
			NCT02365597	Urothelial cancer	II	Active, not recruiting
			NCT03238196	Metastatic breast cancer	I	Recruiting
			NCT04172675	Urinary bladder neoplasms	II	Not yet recruiting
PRN-1371		Pan-FGFRs inhibitor	NCT02608125	Solid tumors	I	Active, not recruiting
ASP5878		Pan-FGFRs inhibitor	NCT02038673	Solid tumors	I	Completed
BLU-9931		FGFR4 (irreversible)	NO			
BLU-554		FGFR4	NCT02508467	HCC	I	Active, not recruiting
			NCT04194801	HCC	I/II	Not yet recruiting
FGF401		FGFR4 (reversible)	NCT02325739	HCC	I/II	Completed
H3B-6527		FGFR4	NCT03424577	Healthy participants	I	Completed
			NCT02834780	HCC	I	Recruiting
INCB062079	UNKNOW	FGFR4 (irreversible)	NCT03144661	HCC	I	Recruiting
				Cholangiocarcinoma		
				Esophageal cancer		
				Nasopharyngeal cancer		
				Ovarian cancer		
				Solid tumors		

According to Figure 1, we had already known that the abnormal expression of FGFR4 occurs obviously in cholangiocarcinoma and liver cancer. In fact, since the occurrence and development of liver cancer are more dependent on FGFR4, the current FGFR4 inhibitors are mainly aimed at the treatment of HCC (Lu et al., 2019). Cholangiocarcinoma is more commonly treated with FGFR2 inhibitors (Raggi et al., 2019). BLU-9931 is an irreversible kinase inhibitor that acts powerfully on FGFR4 but exhibits no sensitivity to other FGFRs, indicating promising kinase group selectivity (Lang et al., 2019). BLU-9931 has the potential to be used as an FGFR4-selective inhibitor to treat HCC patients with FGFR4 signaling abnormalities for the first time (Hagel et al., 2015). BLU-554 is a highly selective kinase inhibitor that inhibits FGFR4 with an IC50 of 5 nM; in contrast, the IC50 range for FGFR1–3 is 624–2,203 nM (Sarker et al., 2016). BLU-554 is currently being tested in ongoing clinical trials to treat HCC (NCT02508467, NCT04194801, etc.). FGF401 is a novel reversible covalent kinase inhibitor that is highly efficient and specific to FGFR4 while having little effect on the other FGFR members and other kinases in the kinome (Weiss et al., 2019). A clinical trial with FGF401 (NCT02325739) for HCC and other solid malignancies is now complete. Joshi et al. used a structure-guided drug design to create H3B-6527, a novel inhibitor selectively and covalently bound to FGFR4. A series of PDX models revealed that H3B-6527 has a beneficial therapeutic effect on patients with overexpression of FGF19, and clinical trials are currently being conducted (NCT03424577, NCT02834780, etc.). INCB062079 is a useful and discriminating irreversible inhibitor targeting FGFR4 (>250-fold vs. FGFR1/2/3) that suppresses the proliferation of HCC driven by increased expression of FGF19 (Ruggeri et al., 2017). Toxicological experiments are currently underway to investigate the safety and tolerability of INCB062079 in patients with a variety of malignancies (NCT03144661).

Although the current task of advancing our knowledge and the use of small-molecule FGFR4 inhibitors is highly interdisciplinary, inhibitors against FGFR4 must be carefully evaluated based on the clinical data. At present, only a few single-agent FGFR4 inhibitors have been confirmed to be efficient for therapy (Lu et al., 2019). Improving our understanding of the pathogenesis that occurs in tumors that overexpress FGFR4, as well as the elucidation of elements that alter the sensitivity to endurance of FGFR4 inhibitors, is vital for a more accurate selection of patients and for increasing the success rate of cancer treatment with FGFR4 inhibitors (Knights and Cook, 2010).

## Conclusion

Many investigations and studies have demonstrated that the FGF19/FGFR4 pathway influences cells’ growth, development, and their differentiation in tumors (Katoh, 2016). Abnormal gene expression of FGFR4 with its ligand FGF19 has been determined as a vital factor in tumor growth (Babina and Turner, 2017).

The research on FGFR4 has focused on the exploitation of small molecular inhibitors (Mellor, 2014). Herein, we reviewed various inhibitors of FGFR4 in the cancer microenvironment, including immune evasion, paracrine signaling, and angiogenesis (Repana and Ross, 2015). However, the effectiveness of FGFR4 inhibitors is still being challenged (Wang et al., 2017). Compared to other RTKs, the selectivity of FGFR4 remains relatively new, and sometimes it needs to be used in combination with other adjuvant drugs in clinical treatment to be effective (Jiang et al., 2017). Moreover, their efficacy seems to apply to only a few cancers (Quintanal-Villalonga et al., 2019a). Additional preclinical studies are required to explore FGFR4 inhibitors further and increase their effectiveness during application.

FGFR4 inhibitors have currently attained remarkable potency, and the use of small-molecule inhibitors remains a powerful therapeutic approach (Heinzle et al., 2014). Multiple FGFR4 inhibitors can be applied to treat cancers where FGFR4 signaling is responsible for tumor development. Besides, the use of FGFR4 inhibitors remains a practical approach in cancer patients with high FGFR4 expression. Selective FGFR4 inhibitors are advantageous because of their low toxicity and their ability to be combined with other treatments for more optimal results (Quintanal-Villalonga et al., 2019b). Although FGFR4-based therapy is still relatively new, it should be completely utilized for treating human diseases. Various modern developments are required to explain FGFR4 biology and its treatment strategy further.

Cancer treatments' resistance to carcinogenic drivers remains a major clinical challenge (Zhao et al., 2016a; Zheng et al., 2019). Various mechanisms may induce this phenomenon, but the consequence is unavoidably relative to signaling reactions that promote cell antiapoptosis and survival (Gatenby and Brown, 2018; Yang et al., 2019). Most kinase inhibitors are hydrophobic with relatively small molecular weights, and they competitively bind to the ATP domain of the related kinase to cause inhibition. A typical resistance mechanism is that the kinase domain has a mutation that blocks the drug from combining with the active site (Jiao et al., 2018; Zhao et al., 2020). It is this mechanism that limits the application of FGFR4 inhibitors. One study found that the FGFR4 gene structure had been altered in patients who develop drug resistance to FGFR4 inhibitors in HCC (Hatlen et al., 2019). These changes were later confirmed by *in vitro* and *in vivo* assays. In the case of continuous dependence on oncogenes, the concept of differential resistance is of great significance for cancer treatment and contributes to the clinical progress of a new generation of FGFR4 inhibitors, which considers resistance mechanisms while maintaining selectivity for FGFR4.
